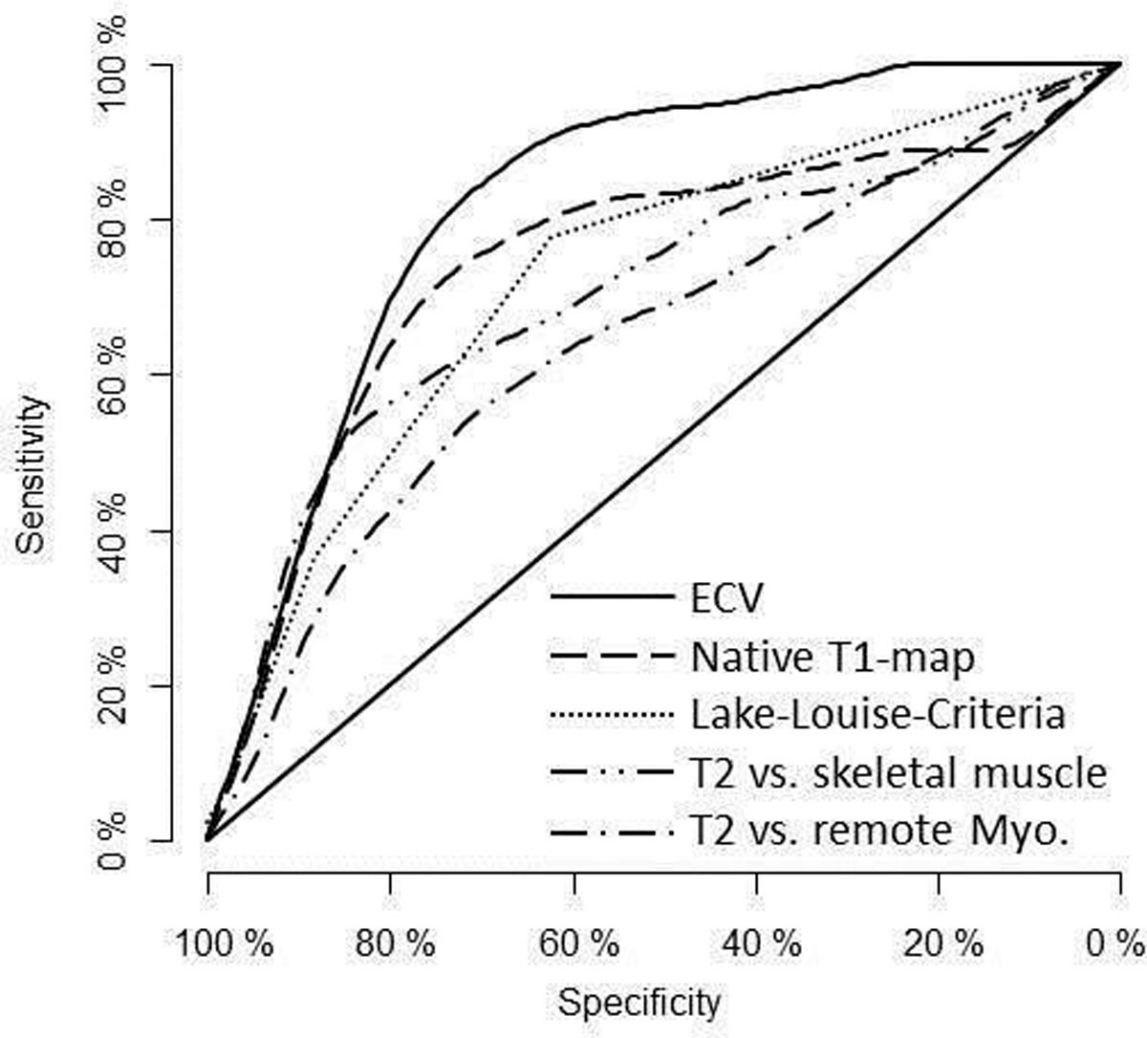# Performance of native and contrast enhanced T1 mapping to detect myocardial damage in patients with suspected myocarditis: A head to head comparison of different CMR-techniques

**DOI:** 10.1186/1532-429X-17-S1-O86

**Published:** 2015-02-03

**Authors:** Jonathan Nadjiri, Eva Hendrich, Albrecht Will, Cornelia Pankalla, Nerejda Shehu, Stefan Martinoff, Martin Hadamitzky

**Affiliations:** 1 Radiologie, Deutsches Herzzentrum München, Munich, Germany

## Background

The diagnosis of acute myocarditis is described to be challenging. Novel techniques like native and contrast enhanced T1 mapping have proven to be superior to standard sequences for differentiation between healthy individuals and acute myocarditis, but a comparison in the clinical setting is not yet done.

We sought to assess the diagnostic performance of native and contrast enhanced T1 mapping compared to T2w-imaging, Relative Enhancement (RE) in T1w as well as Late-Gadolinium-Enhancement (LGE).

## Methods

We investigated 284 patients undergoing CMR examination with suspected myocarditis by performing additional pre and post contrast T1 mapping sequences. Furthermore T2 w dark blood turbospin echo (TSE) sequences, pre and early post Gd T1 w dark blood TSE sequences and inversion recovery spoiled gradient echo sequences for LGE were utilized for myocardial tissue evaluation. Assessment of T1 relaxation time and ECV values was based on 3 short axis views and 1 long axis view. For T2w-imaging evaluation the T2-signal intensity (SI) ratio of myocardium compared to skeletal muscle was used. Additionally T2-SI of potential lesions was compared to remote myocardium and the standard deviation. Relative Enhancement (RE) was calculated from pre and post Gd administration T1w-imaging.

T2-SI ratio and myocarditis associated LGE pattern, were assessed. Reference standard was an elevated troponin level, and a more than 10-fold elevation was considered relevant (0.14ng/ml).

## Results

Native T1 and ECV showed good association with relevant troponin elevations. AUC was 75% (p = 0.0004) for native T1 with an optimal threshold of 993ms (Specificity: 86%; Sensitivity: 70%) and 83% (p < 0.0001) for ECV with and optimal cutoff of 32.4% (Specificity: 73%; Sensitivity: 90%).

AUC for T2w imaging (T2-SI ratio) was 72% (p = 0.0029) with an optimal cutoff of 2.26 (Specificity: 79%; Sensitivity: 63%).

AUC for T2w imaging (T2-SI compared to remote) was 66 % (p = 0.014) with an optimal cutoff of 1.5 standard deviations above remote (Specificity: 64%; Sensitivity: 68%).

Additionally we found good correlation for native T1 and ECV with the Lake-Louise-Criteria(r = 0.41, p = 0.0001 for native T1 and r = 0.42, p = 0.0001 for ECV).

ECV performed significantly better compared to T2-SI in comparison to remote myocardium (p = 0.037) and showed a trend to be superior to T2-imaging in comparison with skeletal muscle (p=0.85).

## Conclusions

Although ECV and native T1 mapping detect other myocardial alterations besides acute damage, these techniques perform at least equally well in comparison to established CMR-techniques in detecting acute myocardial damage. ECV is superior to comparing T2-SI in the myocardium of remote and lesion and at least comparable with the other established methods for evaluation of acute myocarditis. Considering the potential of T1 mapping supposed to be relatively robust in acquisition, routine T1 mapping might augment the diagnostic value of CMR.

## Funding

N/A.

**Figure 1:**